# A Case of Simultaneous Unilateral Anterior and Posterior Stafne Bone Defects

**DOI:** 10.1155/2015/983956

**Published:** 2015-10-29

**Authors:** Hisashi Ozaki, Shigeo Ishikawa, Kenichirou Kitabatake, Kazuyuki Yusa, Hirohiko Tachibana, Mitsuyoshi Iino

**Affiliations:** Department of Dentistry, Oral and Maxillofacial Plastic and Reconstructive Surgery, Faculty of Medicine, Yamagata University, 2-2-2 Iidanishi, Yamagata 990-9585, Japan

## Abstract

Stafne bone defects (SBDs) are asymptomatic mandibular lingual bone depressions mainly caused by soft tissue inclusions. The most common form of SBDs is posterior; the anterior variant of SBD is relatively uncommon. Although posterior SBD is easily diagnosed by the unique location on radiography, anterior SBD is sometimes misdiagnosed and confused with other pathological entities owing to the location. We report herein a case of simultaneous unilateral anterior and posterior SBDs. In the present case, definitive diagnosis for the anterior mandibular cavity was unclear, as in reported cases. Surgical exploration was thus performed for the lesion in the anterior mandibular cavity. Pathologic examination of the removed tissue showed salivary gland with chronic inflammation. Postoperatively, no functional disturbance has been observed. Management of the posterior SBD was conservative, with radiographic follow-up. To the best of our knowledge, this represents the first report of simultaneous unilateral anterior and posterior SBDs.

## 1. Introduction

Stafne bone defects (SBDs) were first described by Stafne as usually unilateral, asymptomatic, well-defined radiolucent lingual bony defects located around the posterior region of the mandible [1]. Since then, numerous cases of this entity have been reported [2]. SBDs have anterior and posterior variants [3]. The posterior variant is the most well-known, located between the mandibular angle and first mandibular molar below the inferior dental canal [4–6]. On the other hand, anterior SBD is a rare lingual bone depression mostly seen in the mandibular canine-premolar region. Since Richard and Ziskind [7] offered the first description of anterior SBD in 1957, almost 50 cases have been reported in the literature [8]. The cause of lingual bony defect remains controversial. Stafne initially suggested that the occurrence of lingual cavities was developmental, as the defect was occupied by cartilaginous tissue due to bone deposition deficiency [1]. However, pressure of glandular tissues on the lingual cortex is well recognized to cause bony depressions [9]. According to this widely accepted concept, the submandibular salivary glands are responsible for posterior SBD, whereas the sublingual salivary glands cause anterior SBD [10]. Posterior SBD can be readily diagnosed because of the unique location in radiographic examination. However, anterior SBD may sometimes be misdiagnosed and confused with pathologic entities such as traumatic or cystic lesions or tumors of the jaw [3]. We present here the first report of simultaneous unilateral anterior and posterior SBDs.

## 2. Case Presentation

**Figure 1:**
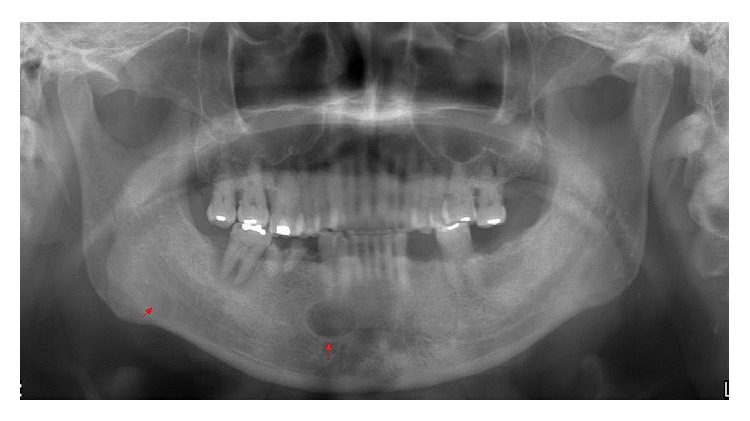
Preoperative panoramic radiography shows two regions of radiolucency in the right mandible, as a well-defined, oval radiolucency just inferior to the premolar root apex, and a slight oval radiolucency at the angle of the mandible below the inferior alveolar canal.

**Figure 2:**
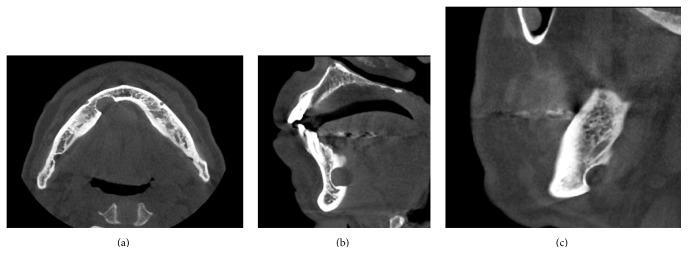
Cone-beam computed tomography (CBCT). (a) Axial image. Two defects of the cortex on the lingual plate of the mandible are apparent. (b) Sagittal image of the cavity in the premolar region, showing dense, radiopaque contents. (c) Sagittal image of the cavity at the angle of the mandible.

**Figure 3:**
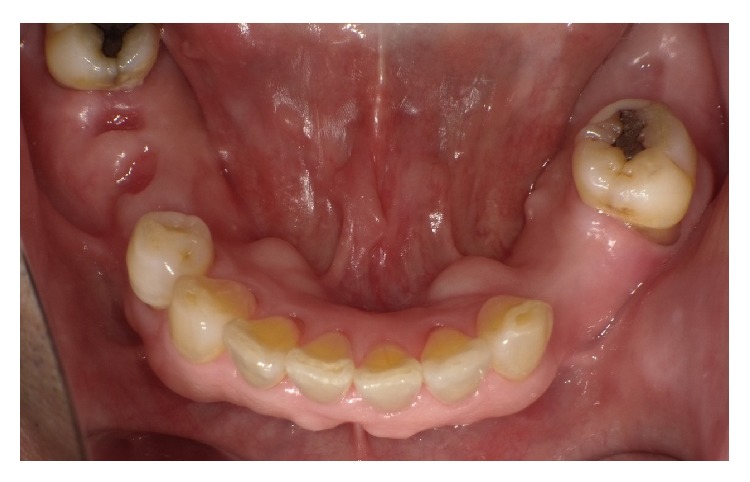
Preoperative intraoral view showing that mucosa overlying the floor of the mouth seems normal.

**Figure 4:**
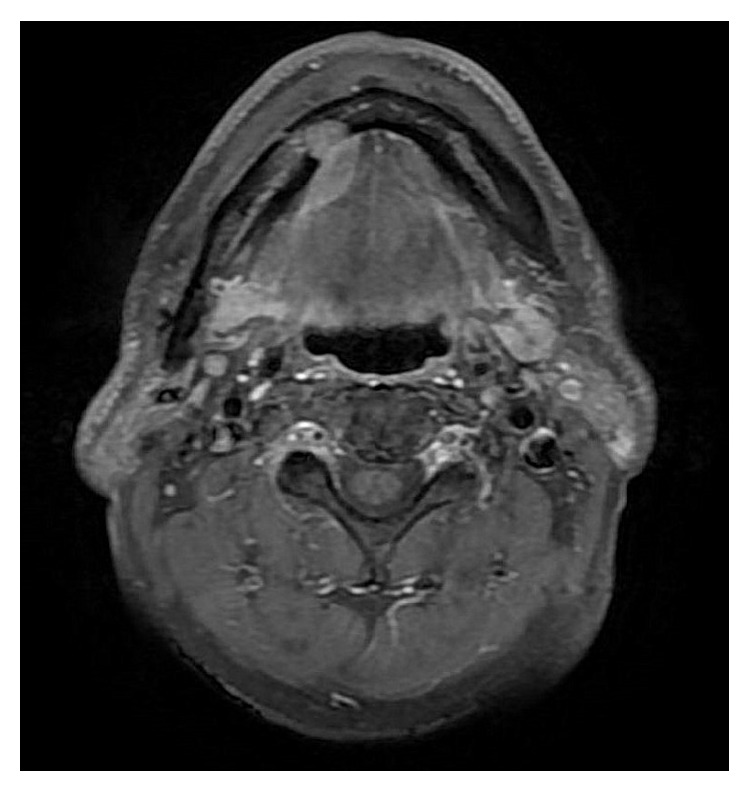
Magnetic resonance imaging (MRI) shows that both bone cavities are filled with soft tissue. The lesion in the premolar region is caulescent from the sublingual gland with identical signal intensity to the right sublingual gland, showing a contrast effect compared to the left sublingual gland. The lesion at the angle of the mandible seems to be part of the submandibular gland.

**Figure 5:**
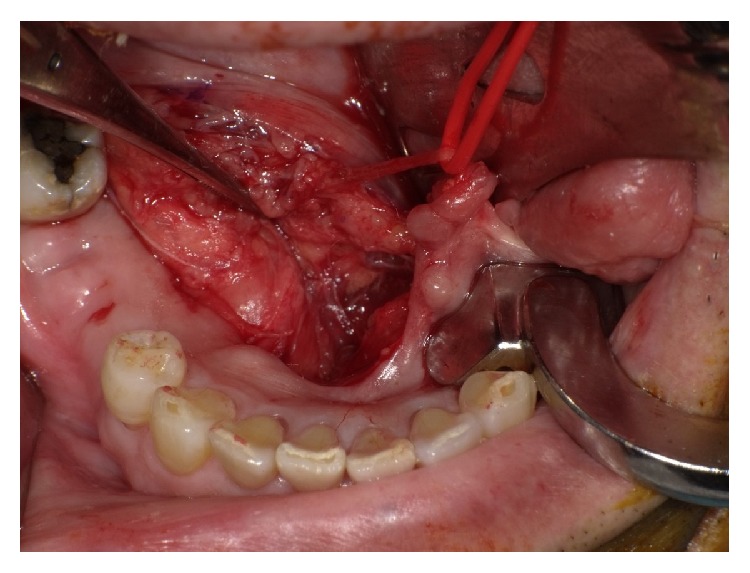
Intraoperative view shows intrusion of a sublingual salivary gland into the cavity.

**Figure 6:**
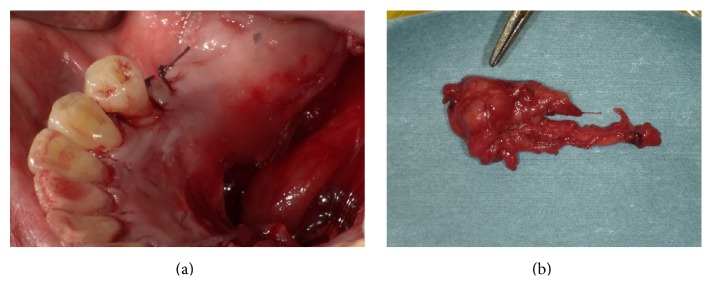
(a) Bone defect after removal of soft tissue in the bone cavity. (b) Surgical specimen. The soft tissue in the bone cavity appears to be part of the sublingual salivary gland.

**Figure 7:**
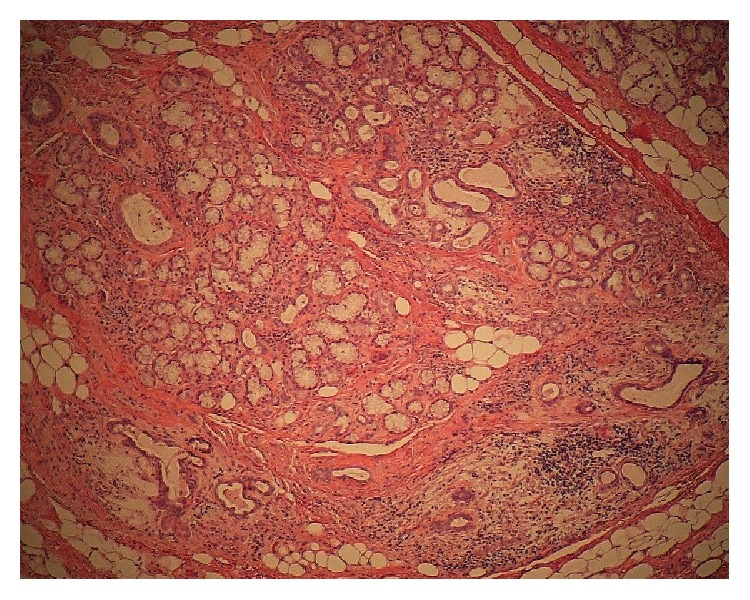
Histopathological examination (original magnification, ×40) reveals expansion of the acinus and duct, retention of mucus, atrophy of the acinus, and lymphocytic infiltration into the lesion of the bone cavity and sublingual gland.

A 76-year-old man was referred to our facility from his family dentist after two cystic lesions were identified in the right mandible. Panoramic radiography revealed two radiolucent areas at premolar and angle regions of the right mandible (Figure 1). Cone-beam computed tomography (CBCT) revealed two defects of the cortex on the lingual aspect of the mandible (Figures 2(a)–2(c)). No discontinuity or erosion was seen in the lingual cortex. On intraoral view, the overlying mucosa of the oral floor was normal (Figure 3). The outflow of saliva from the right sublingual caruncle was scarce, with no discharge of pus. Slight induration was evident when palpating the right floor of the mouth in the premolar region. The tongue showed normal mobility. Magnetic resonance imaging (MRI) with gadolinium contrast showed that both bone cavities were filled with soft tissue. Soft tissue in the mandibular angle seemed to be part of the submandibular gland (Figure 4). In the premolar region, the soft tissue in the cavity showed identical signals to that of the right sublingual gland (Figure 4). The right sublingual gland was mildly enhanced compared to the left sublingual gland. On the basis of these clinical and imaging findings, posterior SBD for the bone cavity in the mandibular angle was diagnosed and the decision was made to provide conservative follow-up by radiographic examination. However, as for the premolar region, not only anterior SBD, but also sublingual sialadenitis was suspected. In addition, sublingual gland tumor could not be completely ruled out. Surgical resection to the soft tissue in the cavity of the premolar region was therefore performed under general anesthesia, including the right sublingual gland. Intraoperatively, intrusion of a sublingual salivary gland into the cavity was seen (Figure 5). No adhesion between the sublingual gland and bone was identified (Figures 6(a) and 6(b)). The pathologic diagnosis was salivary gland with chronic inflammation. Histopathological examination revealed expansion and duct, expansion or atrophy of the acinus in comparison with the acinus to be normal, retention of mucus, and infiltration of lymphocytes into the sublingual gland (Figure 7). Based on these findings, a definitive diagnosis of anterior SBD was made for the lesion in the premolar region. The postoperative course was uneventful, and no functional disturbance has since been observed.

## 3. Discussion

Posterior SBD is the most common variant of SBD, and anterior SBD is rare, with reported prevalences of 0.10–0.48% [11, 12] and 0.009–0.03% [4, 13], respectively. Less common locations include the ascending ramus of the mandible [14]. Most SBDs are unilocular, one on the same side of the mandible, well-defined radiolucencies appearing unilaterally on panoramic radiography, and bilateral, multilocular cases are less frequent [15]. To date, 2 cases exhibiting multilocular appearance of posterior SBD [16, 17] and 6 cases of jaws with bilateral lesions [6, 15, 18–21] have been reported. However, to the best of our knowledge, no cases of simultaneous unilateral anterior and posterior SBDs have previously been reported, so the present case represents the first description of an extremely rare pathology.

Anterior SBD is considered difficult to diagnose, in contrast to posterior SBD [22]. Approximately 50 cases of anterior SBD have been reported, with surgical exploration or biopsy performed in most cases before diagnosis [8]. This is because anterior SBD may be located between or below the tooth roots, so anterior SBD may be misdiagnosed as other radiolucencies, such as odontogenic cysts, various benign tumors, or bone metastases [8]. In the present case, CBCT showed that the lesions were located inside the mandible and pressed on the lingual cortex. From this finding, jaw bone cysts and tumors and bone metastases were excluded from the differential diagnoses. Segev et al. [2] emphasized the usefulness of MRI in making a diagnosis of SBD, because of the superior soft tissue contrast. MRI in the present case showed that the soft tissue in the bony defect was continuous and identical in signal intensity with the sublingual gland. FNAB (fine-needle aspiration biopsy) is diagnostic procedure used to investigate superficial lumps or masses. FNAB is very safe, minor surgical procedure. However, in the present case, the lesion in the bone defect was small and seemed to be difficult to puncture correctly. Therefore, we did not do FNAB for the lesion. Instead of the FNAB, we performed intraoperative frozen section diagnosis and diagnosed salivary gland with chronic inflammation. Differential diagnoses therefore included sublingual gland tumor, sublingual sialadenitis, or anterior SBD. Surgery is not generally considered necessary for the treatment of SBD [3]. However, a review of the literature reported that these cavities can have contents other than glandular tissue, such as fibrous connective tissue, adipose tissue, muscle, nerve, lymph nodes, and blood vessels [23]. Surgical exploration or biopsy should thus be performed to rule out other pathological entities when the diagnosis is uncertain or clinical symptoms are present. We reported an extremely rare case of simultaneous unilateral anterior and posterior SBDs. Accumulation of further cases is needed to clarify the optimal management of SBD.

## References

[1] Stafne E. C. (1942). Bone cavities situated near the angle of the mandible. *The Journal of the American Dental Association*.

[2] Segev Y., Puterman M., Bodner L. (2006). Stafne bone cavity—magnetic resonance imaging. *Medicina Oral, Patología Oral y Cirugía Bucal*.

[3] Dereci Ö., Duran S. (2012). Intraorally exposed anterior Stafne bone defect: a case report. *Oral Surgery, Oral Medicine, Oral Pathology and Oral Radiology*.

[4] Philipsen H. P., Takata T., Reichart P. A., Sato S., Suei Y. (2002). Lingual and buccal mandibular bone depressions: a review based on 583 cases from a world-wide literature survey, including 69 new cases from Japan. *Dentomaxillofacial Radiology*.

[5] Slasky B. S., Bar-Ziv J. (1996). Lingual mandibular bony defects: CT in the buccolingual plane. *Journal of Computer Assisted Tomography*.

[6] Grellner T. J., Frost D. E., Brannon R. B. (1990). Lingual mandibular bone defect: report of three cases. *Journal of Oral and Maxillofacial Surgery*.

[7] Richard E. L., Ziskind J. (1957). Aberrant salivary gland tissue in mandible. *Oral Surgery, Oral Medicine, Oral Pathology*.

[8] Turkoglu K., Orhan K. (2010). Stafne bone cavity in the anterior mandible. *Journal of Craniofacial Surgery*.

[9] Tsui S. H., Chan F. F. (1994). Lingual mandibular bone defect: case report and review of the literature. *Australian Dental Journal*.

[10] de Courten A., Küffer R., Samson J., Lombardi T. (2002). Anterior lingual mandibular salivary gland defect (Stafne defect) presenting as a residual cyst. *Oral Surgery, Oral Medicine, Oral Pathology, Oral Radiology, and Endodontics*.

[11] Layne E. L., Morgan A. F., Morton T. H. (1981). Anterior lingual mandibular bone concavity: report of case. *Journal of Oral Surgery*.

[12] Ström C., Fjellström C. (1987). An unusual case of lingual mandibular depression. *Oral Surgery, Oral Medicine, Oral Pathology*.

[13] Langlais R. P., Cottone J., Kasle M. J. (1976). Anterior and posterior lingual depressions of the mandible. *Journal of Oral Surgery*.

[14] Barker G. R. (1988). A radiolucency of the ascending ramus of the mandible associated with invested parotid salivary gland material and analogous with a stafne bone cavity. *British Journal of Oral and Maxillofacial Surgery*.

[15] Silva P. H., Sindermann D. B., Rondanelli B. M. (2006). Giant mandibular bone defect: report of a case. *Journal of Oral and Maxillofacial Surgery*.

[16] Etöz M., Etöz O. A., Şahman H., Şekerci A. E., Polat H. B. (2012). An unusual case of multilocular stafne bone cavity. *Dentomaxillofacial Radiology*.

[17] Miloğlu Ö., Sekerci A. E., Yasa Y., Dagistan S. (2015). Unilateral bone cavities situated near the angle of the mandible. *Journal of Craniofacial Surgery*.

[18] Smith M. H., Brooks S. L., Eldevik O. P., Helman J. I. (2007). Anterior mandibular lingual salivary gland defect: a report of a case diagnosed with cone-beam computed tomography and magnetic resonance imaging. *Oral Surgery, Oral Medicine, Oral Pathology, Oral Radiology and Endodontology*.

[19] Friedman J. (1964). Ectopic sublingual glands. *Oral Surgery, Oral Medicine, Oral Pathology*.

[20] Miller A. S., Winnick M. (1971). Salivary gland inclusion in the anterior mandible: report of a case with a review of the literature on aberrant salivary gland tissue and neoplasms. *Oral Surgery, Oral Medicine, Oral Pathology*.

[21] Mizuno A., Kawabata T., Nakano Y., Motegi K. (1983). Lingual mandibular bone defect—idiopathic bone cavity. Report of a case. *International Journal of Oral Surgery*.

[22] Taysi M., Ozden C., Cankaya B., Olgac V., Yildirim S. (2014). Stafne bone defect in the anterior mandible. *Dentomaxillofacial Radiology*.

[23] Quesada-Gómez C., Valmaseda-Castellón E., Berini-Aytés L., Gay-Escoda C. (2006). Stafne bone cavity: a retrospective study of 11 cases. *Medicina Oral, Patología Oral y Cirugía Bucal*.

